# Potential Ameliorating Effects of Fluvoxamine in a Rat Model of Endotoxin-Induced Neuroinflammation: Molecular Aspects Through SIRT-1/GPX-4 and HMGB-1 Signaling

**DOI:** 10.1007/s12035-025-04764-1

**Published:** 2025-02-15

**Authors:** Muhammet Yusuf Tepebaşı, Halil Aşcı, Pınar Aslan Koşar, Emine Nur Dinçer, Esma Selçuk, Öznur Kolay, İbrahim Hüseynov

**Affiliations:** 1 Department of Genetics, Faculty of Medicine, Suleyman Demirel University, Isparta, Turkey; 2 Department of Pharmacology, Faculty of Medicine, Suleyman Demirel University, Isparta, Turkey; 3 Department of Medical Biology, Faculty of Medicine, Suleyman Demirel University, Isparta, Turkey; 4 Department of Pathology, Faculty of Veterinary Medicine, Mehmet Akif Ersoy University, Burdur, Turkey; 5 Institute of Health Sciences, Department of Pharmacology, Suleyman Demirel University, Isparta, Turkey; 6 Faculty of Medicine, Suleyman Demirel University, Isparta, Turkey

**Keywords:** Brain, Cerebellum, Hippocampus, Lipopolysaccharide, Oxidative stress, SSRI

## Abstract

Research on the tissue-protective effects of fluvoxamine (FLV), a selective serotonin reuptake inhibitor, rapidly expands. This study explores FLV’s potential to protect against lipopolysaccharide (LPS)-induced neuroinflammation, a key factor in systemic inflammation-related neuronal damage. Four equal groups of thirty-two female Wistar Albino rats were created: FLV, LPS-FLV (50 mg/kg intraperitoneal), LPS (5 mg/kg intraperitoneal), and control. Both drugs were given in one dose on the same day. Tissues from the brain cortex, cerebellum, and hippocampus were taken for histopathology, immunohistochemistry, biochemistry, and genetic analysis. In the LPS group, histological examinations revealed hyperemia, edema, mild degeneration, neuronal death, and modest gliosis. Additionally, while apelin and total antioxidant status levels were reduced, greater levels of oxidative stress index, glial fibrillary acidic protein (GFAP), mammalian target of rapamycin (mTOR), and total oxidant status were noted. FLV treatment reversed all these findings. Genetic analyses revealed that LPS decreased sirtuin-1 (SIRT-1) and glutathione peroxidase 4 (GPX-4) while increasing high mobility group box protein 1 (HMGB-1). FLV treatment reversed all these parameters, and a significant result was obtained only with GPX-4. In this study, FLV treatment was shown to have anti-inflammatory and neuroprotective effects through various mechanisms on the brain cortex, cerebellum, and hippocampus tissues in addition to its antidepressant effects.

## Introduction

An inflammatory response in the central nervous system known as neuroinflammation can result from several pathogenic events, including infection, trauma, ischemia, and toxicity [1]. Furthermore, dysregulation of immune and inflammatory responses may contribute to microglial activation, leading to neuroinflammation and the release of harmful substances such as cytokines and nitric oxide [2].

Phosphorylation of the mammalian target of the rapamycin (mTOR) pathway is an important factor in microglia activation [3]. Nuclear factor kappa beta (NF-kB) activity is raised when the mTOR pathway is engaged, which increases the expression of inflammatory molecules [4]. Sirtuin 1 (SIRT-1), a neurosteroid hormone released by the cerebellum and pineal gland, inhibits the NF-kB pathway at various stages of the inflammatory process [5]. In addition, SIRT-1 can lower oxidative stress by increasing antioxidant enzymes [6].

Increased inflammation is also associated with increased expression of GFAP, the main filament of mature astrocytes in the central nervous system [7]. Reactive astrogliosis is caused by NF-kB binding to the DNA promoter region. Injured brain tissue may sustain further damage as a result of pro-inflammatory and cytotoxic cytokines released by hyperactive astrocytes that damage neurons or oligodendrocytes. Investigations have indicated that GFAP levels rise during astrogliosis [8].

Recent evidence has shown that apelin-13, an active form of apelin, suppresses neuroinflammation by inhibiting microglia and astrocyte activation and improves cognitive decline in various pathological processes [9].

High mobility group box protein 1 (HMGB-1) is a ubiquitous nuclear protein released by glia and neurons during activation of inflammation [10]. HMGB-1 activates sterile inflammation by binding to advanced glycation end products and toll-like receptor-4, causing macrophages and endothelial cells to produce TNF-α, IL-1, and IL-6. This binding also activates the NF-kB pathway and facilitates the upregulation of HMGB-1 and the expression of pro-inflammatory mediators. Animal experiments have shown that blocking HMGB-1 suppresses neuroinflammation and provides neuroprotective effects [11].

Initially identified as phospholipid hydroperoxide glutathione peroxidase, the selenoprotein glutathione peroxidase 4 (GPX-4) is the primary oxidoreductase that uses glutathione as a reducing agent to scavenge lipid peroxidation products [12].

A common selective serotonin reuptake inhibitor (SSRI) used to treat anxiety and depression is fluvoxamine (FLV). Recent studies have highlighted the anti-inflammatory effects of FLV. According to a study, FLV prevented the lesion’s toxic consequences and markedly reduced inflammation by preventing microglia activation in the striatum, which typically results in an increase in reactive oxygen species generation in a rat model of Parkinson’s disease linked to depression [13]. Therefore, elucidating the potential protective mechanisms of agents that act on the central nervous system against neuroinflammation is of significant importance.

The purpose of this study is to look into and understand the potential protective benefits of FLV treatment on brain tissue damage caused by neuroinflammation.

## Materials and Methods

### Animals and Experimental Design

The study included thirty-two female, approximately 300 g Wistar Albino-type rats, divided into four groups (each containing eight rats), which were obtained from SDU Experimental Animal Laboratory. Groups:

1. Control group: 30 min after the rats’ left inguinal region received 0.5 ml of saline (SF), the right inguinal region received 0.5 ml of saline intraperitoneally (i.p.).
2. 2-LPS group: 30 min after giving the rats 0.5 ml of LPS (L2630-100 mg, Sigma-Aldrich, USA) intraperitoneally at a dose of 5 mg/kg, the rats received 0.5–1 ml of SF intraperitoneally (i.p.) in the left inguinal region [14]. Solid LPS dissolved in SF.
3. 3-LPS + FLV group: 50 mg/kg FLV (Faverin 100 mg, Abbot Ltd., Turkey) in 0.5–1 ml volume in SF was injected i.p. to the rats’ left inguinal area, followed by 5 mg/kg LPS in 0.5 ml volume i.p. to the right inguinal region 30 min later [15].
4. 4-FLV group: FLV at a dose of 50 mg/kg in 0.5–1-ml volume of SF was injected i.p. in the rats’ left inguinal area, followed by 0.5 ml SF i.p. in the right inguinal region 30 min later.

The animals were put to death under anesthesia with 90 mg/kg ketamine (Keta-Control, Doğa İlaç, Turkey) and 8–10 mg/kg xylazine (Xylazinbio 2%, Bioveta, Czech Republic) 6 h after the previous medication administration. Euthanasia was carried out via surgical exsanguination using inferior vena cava blood after the abdominal incision. Ten percent formaldehyde was used to preserve half of the extracted brain, cerebellar, and hippocampus tissues for use in immunohistochemical and histopathological examinations. The remaining portions of brain tissues were divided in half and kept at − 80 °C for biochemical and genetic examinations.

### Histopathological Examination

The brain, hippocampus, and cerebellar tissues were carefully removed during the necropsy and stored in 10% buffered formalin for histological examination. Following that, a fully automated tissue processor was used to process the tissues as part of standard procedures. The paraffin blocks were sectioned using a rotary microtome (Leica RM2155, Leica Microsystems, Wetzlar, Germany) that measured 5 µm in thickness. The sections were deparaffinized, cleaned with xylene, stained with hematoxylin–eosin (HE), and then rehydrated with ethanol at decreasing concentrations before being covered. A light microscope was used to evaluate the histological alterations. The histological lesions in several brain areas were evaluated semiquantitatively. The extent of gliosis, neuronal damage, hyperemia, and bleeding were assessed for this. Scores ranging from 0 to 3 were assigned to the descriptions based on their harshness. Table 1 displays the scoring methodology for the histopathological results [16].

**Table 1:** An explanation of the histologic scores

		Histopathological evaluation	Immunohistochemical evaluation
Score	0	No lesion	A negative stain
	1	Lesions in < 20% of the fields	Diffuse staining
	2	Lesions in 20–60% of the fields	Weak staining
	3	Lesions in all fields	Severe staining

### Immunohistochemical Examination

Apelin (Rabbit Anti-Apelin polyclonal antibody (bs2425R)), mTOR (mTOR (Ser2448) Polyclonal Antibody (bs-3494R)), and GFAP (GFAP (3F4) Monoclonal Antibody (bs-3494R)) were used to immunostain sections placed onto poly-L-lysine slides utilizing the method of streptavidin biotin peroxidase. Every primary antibody was utilized at a dilution of 1/100. After sections were exposed to primary antibodies for a full night, they were immunohistochemically stained using biotinylated secondary antibodies and streptavidin–alkaline phosphatase conjugate. A ready-to-use commercial kit called the EXPOSE Mouse and Rabbit Specific HRP/DAB Detection IHC kit (ab80436), from Abcam in Cambridge, UK, was used to obtain the secondary antibody and chromogen. The antibody dilution solution was used as a stand-in for the primary antiserum step in the negative controls. Every examination was conducted using blinded samples by a professional histopathologist from a different university.

The proportion of cells that were positively immunostained for each marker in 10 distinct fields for each slide for all groups was computed at an objective magnification of 40 × . The image analyzer’s output was counted using the ImageJ program (National Institutes of Health, Bethesda, MD, version 1.48). The photos were cropped and separated into color channels, and any artifacts were eliminated before counting. Cells inside the regions of interest were tallied using the software’s counting tool once they had been selected using a selection tool. Positive staining was identified by its brown color, and only cells exhibiting significant brown staining were deemed positive. Using the Database Manual Cell Sens Life Science Imaging Software System (Olympus Co., Tokyo, Japan), microphotographs were captured. The scoring system for the histological and immunohistochemical results is shown in Table 1 [16].

### Biochemical Analysis

Brain tissues taken for oxidative stress assessments were sectioned and placed in Eppendorf tubes. They were then stored at − 20 °C until the day of analysis. Then, it was homogenized in phosphate buffer pH 7.4. Following homogenization, the samples were centrifuged (Nuve NF 1200R, Ankara, Turkey) at 2000 rpm/20 min. Measurements were made by the spectrophotometric method. Analysis of the total antioxidant status (TAS) and total oxidant status (TOS) levels was done to determine the degree of oxidative stress. OSI value was calculated with the TOS/TAS/10 formula [17].

### Reverse Transcription-Polymerase Chain Reaction (RT-qPCR)

RNA was extracted from homogenized brain tissues according to the manufacturer’s instructions and the GeneAll RiboEx (TM) RNA Isolation Kit (Cat No. 301–001) (GeneAll Biotechnology, Seoul, Korea). The amount and purity of the extracted RNAs were measured using a BioSpec-nano NanoDrop. The cDNA Synthesis Kit (Cat No. C03-01–05) was used to undertake cDNA synthesis in accordance with the instructions. The Primer-BLAST tool on the NCBI website was used to identify a few mRNA sequences, and then potential primer sequences were looked at. Table 2 lists the accession numbers, product sizes, and primer sequences that were employed. The SYBR Master Mix (Cat No. Q04-01–05) was utilized in a Biorad CFX96 real-time PCR apparatus (CA, USA) to measure the gene expression levels. The Rn18s were employed as a housekeeping gene in the investigation. The reaction mixture was produced to a final volume of 20 µL following the manufacturer’s instructions. Following the kit’s manufacturer’s instructions, the reaction mixture was added to a real-time qPCR apparatus with a thermal cycling setup, and each sample was tested three times. After normalizing the data using the 2^−ΔΔCt^ technique, relative mRNA levels were determined.

**Table 2:** Gene accession numbers, product sizes, and primary sequences

Genes	Primary sequence	Product size	Accession number
Rn18s (housekeeping)	F: CTCTAGATAACCTCGGGCCG	209 bp	NR_046237.2
	R: GTCGGGAGTGGGTAATTTGC		
SIRT-1	F: GGTAGTTCCTCGGTGTCCT	152 bp	NM_001414959.1
	R: ACCCAATAACAATGAGGAGGTC		
HMGB-1	F: GCGCTTTTGTGATGGAGTGC	244 bp	NM_012963.3
	R: GCACCAAGTGTTGTTAATGGGG		
GPX-4	F: CATTGGTCGGCTGCGTGA	276 bp	NM_017165.4
	R: GGTTTTGCCTCATTGCGAGG		

### Statistical Analysis

In the evaluation of the data, the nonparametric statistical method one way-ANOVA (posthoc Tukey) was preferred because the number of samples in each group was less than 30 and the variances were different. The statistical analyses were all assessed with GraphPad Prism. The group mean + SD was utilized to express the data, and the appropriate analysis types were applied based on the type of data. All analyses were considered significant at a *p*-value of less than 0.05.

## Results

### Histopathological Results

**Fig. 1:**
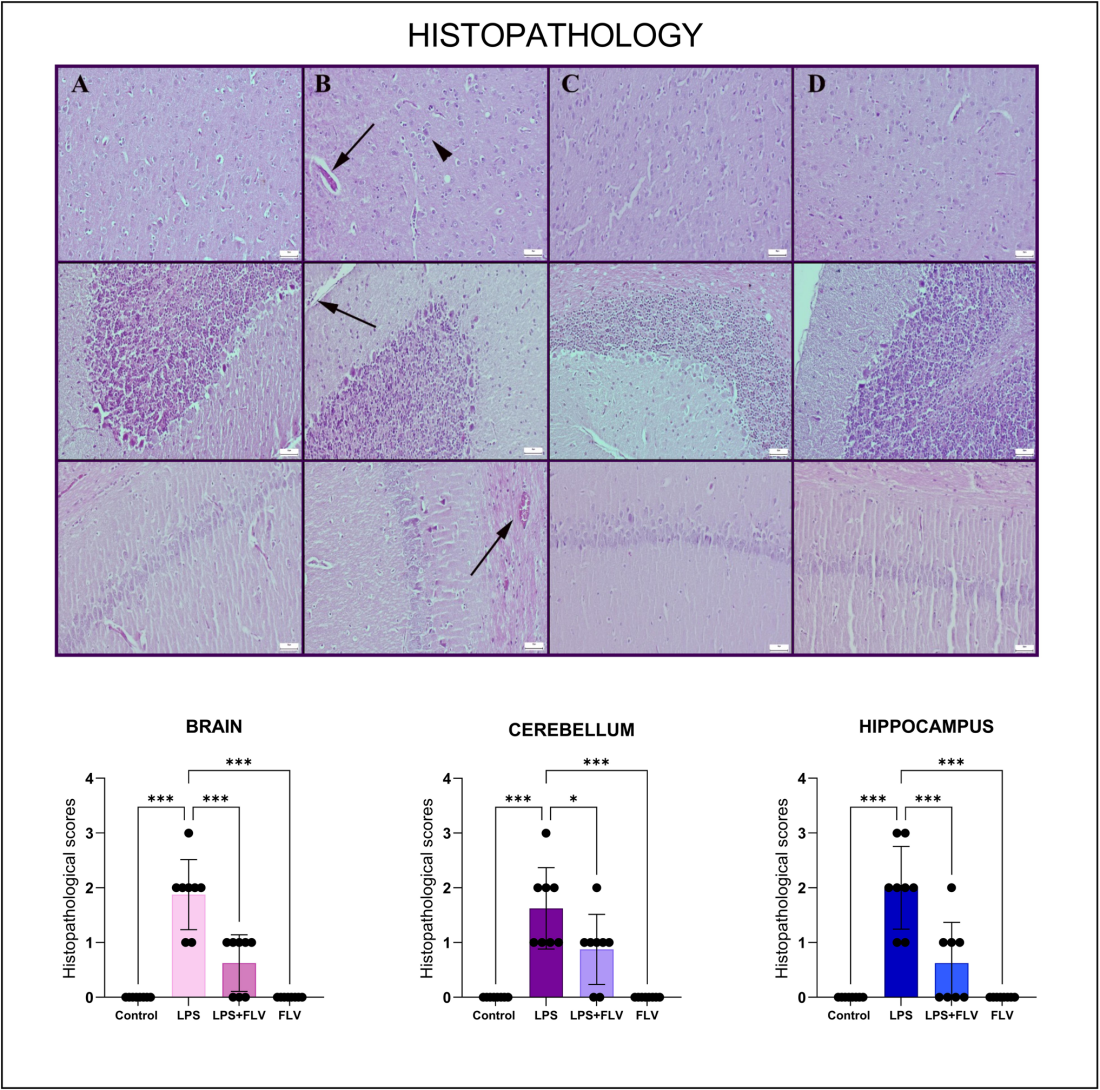
The histopathological differences between the groups in the tissues of the brain (upper row), cerebellum (middle row), and hippocampus (bottom row). (**A**) Normal histological architecture of tissues in rats from the control group (*n* = 8), (**B**) marked hyperemia, edema (arrows), and gliosis (arrowhead) in rats belonging to the LPS group (*n* = 8), (**C**) decreased histopathological lesions in rats in the LPS + FLV group (*n* = 8), (**D**) normal tissue histology in the FLV group (*n* = 8), HE, scale bars = 50 µm. Values are presented as means ± standard deviation. One-way ANOVA (post-hoc Tukey) test was used. *p < 0.05, **p ≤ 0.01 ***p ≤ 0.001

The control group showed no abnormal lesions during the histopathology investigations. However, in the brain, LPS caused considerable hyperemia, edema, mild degeneration, neuronal death, and modest gliosis. In addition, the LPS group frequently experienced hyperemia and degeneration in the cerebellum and hippocampus. Following FLV treatment, the lesions became better (Fig. 1).

### Immunohistochemistry Results

**Fig. 2:**
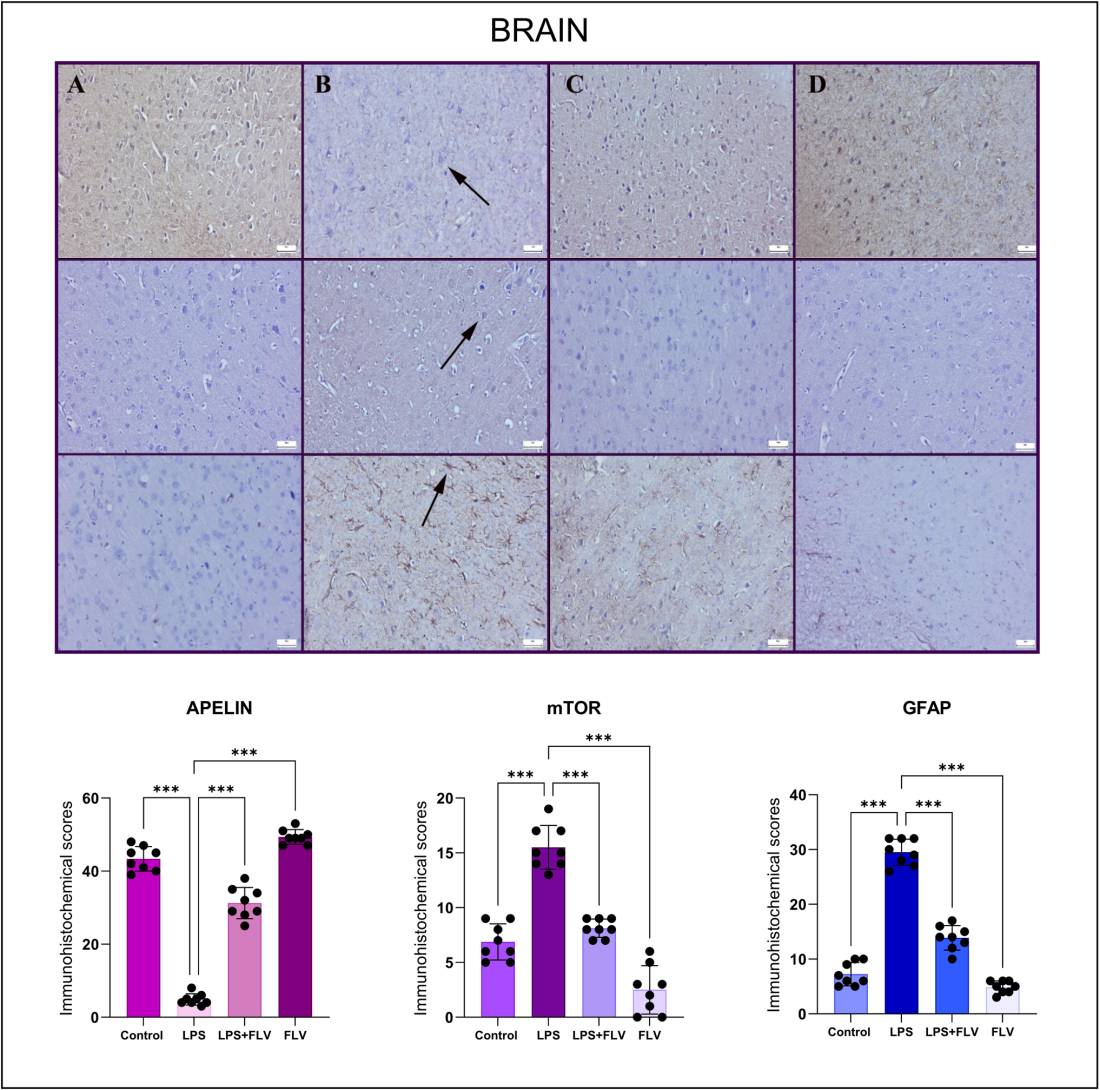
Expressions of apelin (upper row), mTOR (middle row), and GFAP (bottom row) in brain tissues varied among the groups. (**A**) Marked apelin, negative to slight mTOR and GFAP expressions in the control group (*n* = 8), (**B**) decreased apelin, increased mTOR and GFAP of expressions (arrows) in LPS group (*n* = 8), (**C**) increased apelin, decreased mTOR and GFAP of expressions in LPS + FLV group (*n* = 8), (**D**) marked apelin, negative to slight mTOR and GFAP expressions in FLV group (*n* = 8), streptavidin biotin peroxidase method, scale bars = 50 µm. Values are presented as means ± standard deviation. One-way ANOVA (post-hoc Tukey) test was used. *p < 0.05, **p ≤ 0.01, ***p ≤ 0.001

**Fig. 3:**
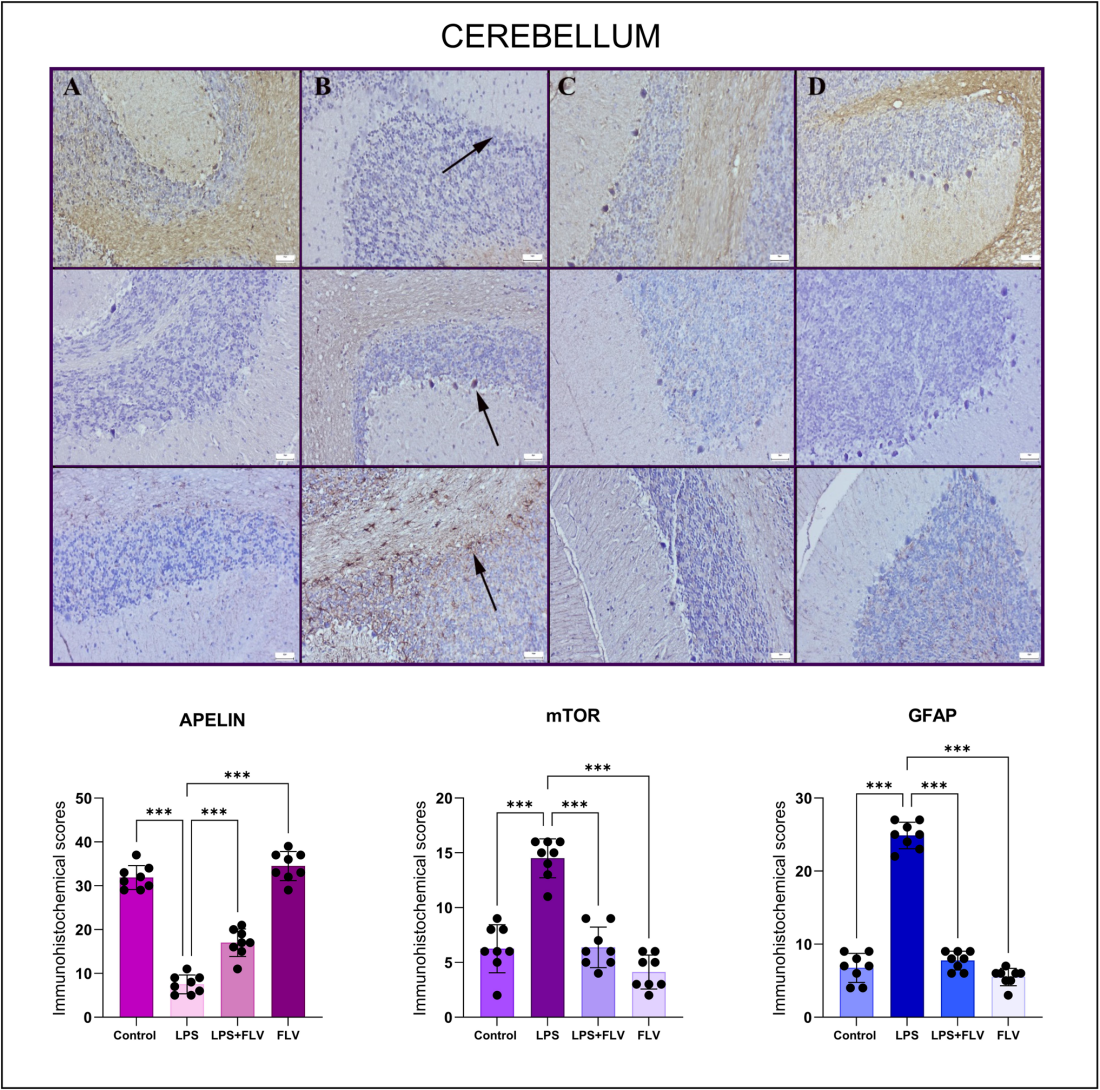
Expressions of apelin (top row), mTOR (middle row), and GFAP (bottom row) in cerebellar tissues varied between the groups. (**A**) Normal apelin, negative to slight mTOR and GFAP expressions in rats belonging to the control group (*n* = 8), (**B**) decreased apelin, increased mTOR and GFAP of expressions (arrows) in neurons in rats among the LPS group (*n* = 8), (**C**) increased apelin, decreased mTOR and GFAP of expressions in rats in the LPS + FLV group (*n* = 8), (**D**) marked apelin negative to slight mTOR and GFAP expressions in rats belonging the FLV group (*n* = 8), streptavidin biotin peroxidase method, scale bars = 50 µm. Values are presented as means ± standard deviation. One-way ANOVA(post-hoc Tukey) test was used. *p < 0.05, **p ≤ 0.01, ***p ≤ 0.001

**Fig. 4:**
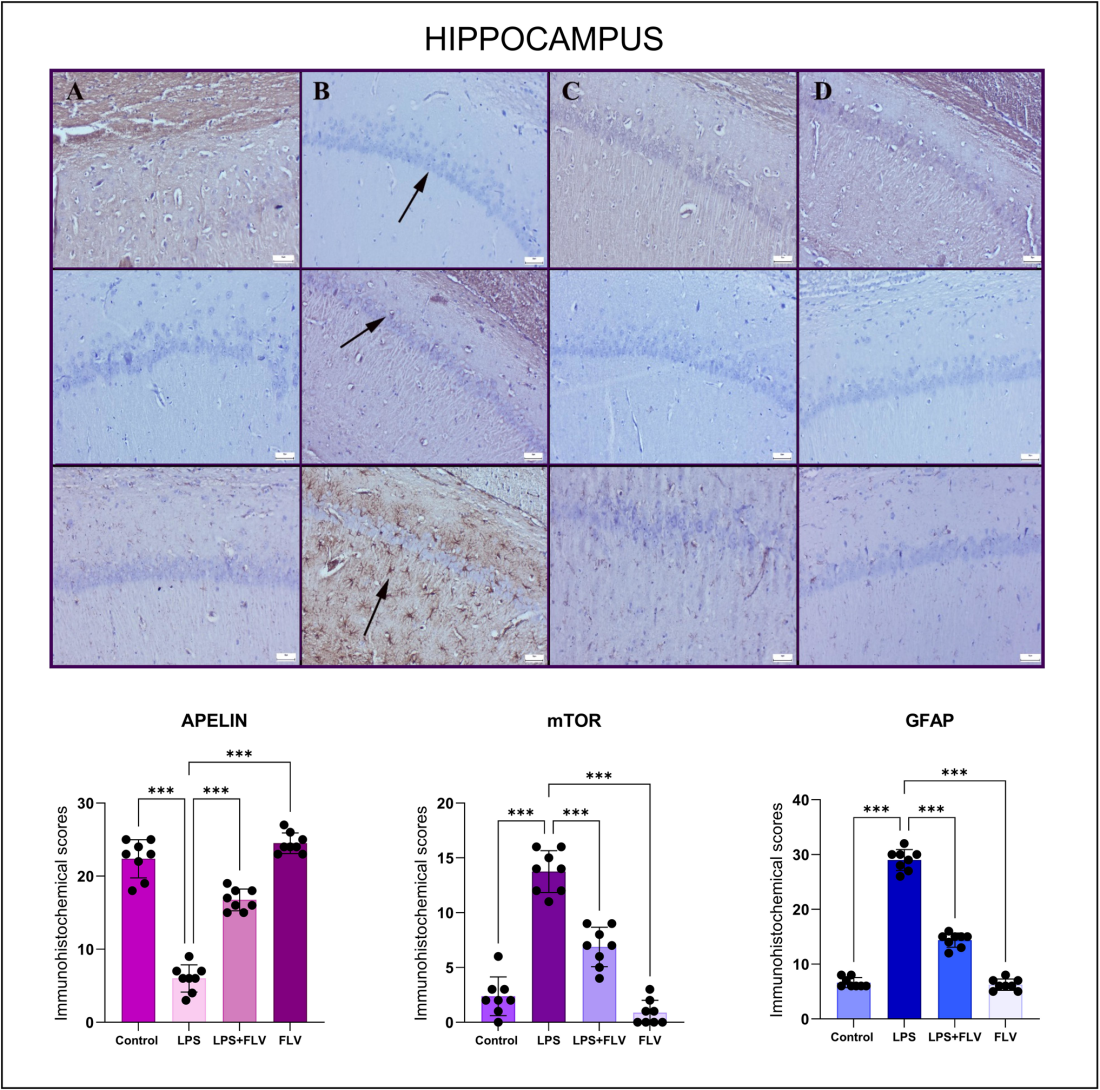
Expressions of GFAP (bottom row), mTOR (middle row), and apelin (top row) in the hippocampal tissues varied throughout the groups. (**A**) Marked apelin, negative to slight mTOR and GFAP expressions in the control group (*n* = 8), (**B**) decrease apelin, increased mTOR and GFAP of expressions (arrows) in the LPS group (*n* = 8), (**C**) increased apelin, decreased mTOR and GFAP of expressions in the LPS + FLV group (*n* = 8), (**D**) marked apelin negative to slight mTOR and GFAP expressions in the FLV group (*n* = 8), streptavidin biotin peroxidase method, scale bars = 50 µm. Values are presented as means ± standard deviation. One-way ANOVA (post-hoc Tukey) test was used. *p < 0.05, **p ≤ 0.01, ***p ≤ 0.001

Apelin expression was shown to be significantly higher in the brain, cerebellum, and hippocampus tissues of the FLV and control groups during the immunohistochemical study; however, the application of LPS led to a significant decrease in expressions in neurons of the central nervous system. In the LPS + FLV group, FLV therapy raised apelin expressions in the brain and, hippocampus, and but not in the cerebellum. The LPS-administered group had considerably higher mTOR and GFAP expressions than the control and FLV groups, which showed negative to modestly raised expressions. The LPS + FLV group’s mTOR and GFAP expressions decreased as a result of FLV therapy (Figs. 2, 3, 4).

### Biochemical Results

**Fig. 5:**
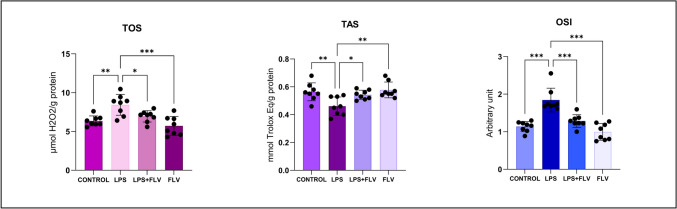
Graph of biochemical values. Statistical analysis of biochemical values performed with one-way ANOVA (post-hoc Tukey). Values are presented as means ± SD. *TAS*, total antioxidant status; TOS, total oxidant status; OSI, oxidative stress index; LPS, lipopolysaccharide; FLV, fluvoxamine. Values are presented as means ± standard deviation. One-way ANOVA (post-hoc Tukey) test was used. *p < 0.05, **p ≤ 0.01, ***p ≤ 0.001

Figure 5 displays the TOS, TAS, and OSI values of the rats analyzed in the study based on the groups. Statistically significant distinctions were noted between the LPS and control groups. The LPS group exhibited significantly greater TOS and OSI values (*p* = 0.002 and *p* ≤ 0.001, respectively) compared to the control group. However, the TAS values were significantly lower (*p* = 0.006) in the LPS group. TAS values were substantially greater (*p* = 0.036) in the LPS + FLV group compared to the LPS group, whereas TOS and OSI values were significantly lower (*p* = 0.034 and *p* ≤ 0.001, respectively) in the LPS + FLV group. These variations indicate that the rats in the FLV groups experienced a greater increase in TAS values than the rats in the LPS group. TAS, TOS, and OSI levels did not differ statistically significantly between the FLV and control groups.

### Genetical Results

**Fig. 6:**
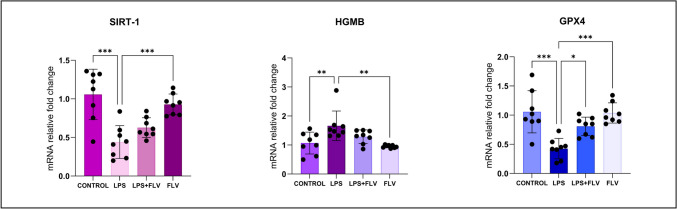
Graph of genetical values. Statistical analysis of biochemical values performed with One-way ANOVA (post-hoc Tukey). Values are presented as means ± SD. SIRT-1, sirtuin 1; HMGB-1, high mobility group box; GPX-4, glutathione peroxidase 4; LPS, lipopolysaccharide; FLV, fluvoxamine. *p < 0.05, **p < 0.01, ***p < 0.001, Error bars represent standard deviation

SIRT-1, HMGB-1, and GPX-4 levels showed statistically significant differences between the LPS and control groups (Fig. 6). While the HMGB-1 values in the LPS group were significantly higher (*p* = 0.008), the GPX4 and SIRT-1 values in the LPS group were significantly lower than those in the control group (*p* ≤ 0.001 for all). The LPS + FLV group showed significantly increased GPX-4 values (*p* = 0.014) when compared to the LPS group, but there were no statistically significant variations in the levels of HMGB and SIRT-1. In the SIRT-1, HMGB-1, and GPX-4 levels, there were no statistically significant differences between the FLV and control groups.

## Discussion

Depression is one of the most prevalent mental illnesses in the modern era, and its mechanism is thought to be caused by inflammation in the brain tissue [18].

Therapeutic agents used in the treatment of this disease are known to exert therapeutic effects by using various mechanisms [19]. Antidepressants are primarily categorized based on their mechanisms of action. Selective serotonin reuptake inhibitors (SSRIs): These medications, including fluoxetine, sertraline, and fluvoxamine, inhibit the reuptake of serotonin (5-HT) into presynaptic neurons, thereby increasing its availability in the synaptic cleft. Serotonin-norepinephrine reuptake inhibitors (SNRIs): Drugs like venlafaxine and duloxetine block the reuptake of both serotonin and norepinephrine, enhancing their synaptic concentrations. Tricyclic antidepressants (TCAs): Examples include amitriptyline and nortriptyline, which inhibit the reuptake of norepinephrine and serotonin but also affect other neurotransmitter systems, leading to a broader side effect profile. Monoamine oxidase inhibitors (MAOIs): Medications such as phenelzine and tranylcypromine inhibit the enzyme monoamine oxidase, responsible for breaking down neurotransmitters like serotonin, norepinephrine, and dopamine.

Especially SSRI drugs, known to affect increasing serotonin concentration in the synaptic gap, are used quite frequently [20]. Sexual dysfunction and sleep disorders that occur during this treatment may limit the use of medication [21]. Nevertheless, proving that such drugs, preferred as an effective treatment method, have regressive effects on inflammation in brain tissue in addition to the main mechanisms in depression will be important in the fight against neuroinflammatory conditions secondary to other diseases [22]. For example, Alzheimer’s and Parkinson’s diseases characterized by neuronal degeneration or brain damage secondary to increased blood–brain barrier permeability due to severe inflammatory conditions developing in the peripheral compartment are associated with depression [23, 24]. While FLV operates as a selective serotonin reuptake inhibitor (SSRI), its neuroprotective mechanisms extend beyond mere serotonin modulation [25]. Unlike antidepressants like rolipram, which function as phosphodiesterase-4 (PDE4) inhibitors to increase cyclic adenosine monophosphate (cAMP) levels, FLV exhibits anti-inflammatory effects by modulating oxidative stress and reducing proinflammatory markers such as HMGB-1 [26, 27]. This mechanism suggests that FLV’s role may involve pathways independent of those utilized by traditional SSRIs or PDE4 inhibitors. Further research comparing FLV’s effects with PDE4 inhibitors or other classes of antidepressants could elucidate whether FLV’s neuroprotective actions are mediated through shared or distinct pathways.

Prooxidant molecules formed in the case of pro-inflammatory and oxidative stress in inflammatory conditions occurring in the damage of non-brain organs pass into the blood and reach the blood–brain barrier and may cause an increase in permeability due to the damage table they create there [28, 29]. It is usual for the brain and cerebellum, which have closed system protection, to be affected by these molecules [30]. It has been proven that these substances, which activate intracellular pathways by binding to their receptors in tissues, stimulate inflammation, oxidative stress, apoptosis, and even autophagy as a result of cellular response. In addition, gliosis in the brain tissue shows that microglia are activated and contribute to the occurrence of the neuroinflammatory picture [28]. In the histopathological data of this study, hyperemia, edema, mild degeneration, neuronal death mild gliosis in the brain tissue, and hyperemia and degeneration in the cerebellum and hippocampus tissue indicate that the inflammatory picture mentioned above has occurred.

Apelin, an endogenous neuropeptide, is associated with neuroprotection, inflammation regulation, and oxidative stress reduction. It is known that neuroprotection provided by increasing the levels of this peptide can reverse the above-mentioned damage mechanisms [31, 32]. In the immunohistochemical analyses of this study, in contrast to the decrease in apelin expression in the damage groups, the increase in the expression of mTOR, which is an indicator of autophagy development, and GFAP, which shows the development of microglia activation, indicates that the mentioned neuroprotection is regressed. In this study, FLV treatment was shown to increase apelin expression while reducing markers of inflammation and oxidative stress such as mTOR and GFAP. These findings suggest that FLV’s ability to upregulate apelin contributes to its neuroprotective effects by countering oxidative stress and inflammatory processes. However, the precise interaction between FLV and apelin signaling remains to be clarified. It is possible that FLV enhances apelin expression either directly or through upstream pathways like SIRT-1 activation [33]. Additional studies are needed to explore whether apelin acts as a mediator or downstream effector of FLV’s protective mechanisms, potentially involving receptor-dependent signaling cascades.

It can be said that the increase in apelin level with FLV treatment is beneficial for the protection of neuronal tissues, but it should be kept in mind that this detected expression may result in a decrease in apelin expression secondary to neuronal protection by acting through other mechanisms. Again, in addition to the increased apelin expressions in all three tissues with FLV treatment, the decrease in mTOR and GFAP expressions proves that this antidepressant has neuroprotective effects. On the other hand, the fact that these effects were observed even with a single dose of FLV used in this study, which is an acute experimental model, and that the apelin expressions in the group using FLV alone were minimally higher than in the control group may be interpreted that increasing doses may increase apelin secretion. In future studies, this situation can be further clarified with studies in which repeated drug administration is performed in a longer experimental model.

The mentioned prooxidant molecules are also known to trigger inflammation through their receptors as well as cause oxidative stress. [28, 34]. In addition to histopathological data, TOS and OSI levels, which are proven to increase in the damage group and indicate the development of oxidative stress, support this information The balance between oxidant and antioxidant substances has been disrupted due to increased oxidation damage. As a result, the antioxidant capacity has become insufficient, and the increases mentioned above have occurred. Decreased TAS values in the damage group may be due to the increase in antioxidant enzyme activity by FLV treatment or may be due to decreased TAS utilization secondary to decreased inflammation. The fact that the TAS values of the drug-only group did not increase considerably when compared to the control group suggests that the usage of antioxidants decreased as a result of the decrease in inflammation, rather than enhancing the drug’s antioxidant enzyme capacity.

SIRT-1 signaling is a crucial mechanism in reducing oxidative stress. It has been demonstrated that raising the expression of this gene promotes nuclear factor erythroid 2-related factor 2 mediated heme oxygenase 1 expression while also providing antioxidant action by increasing GPX-4 synthesis, one of the antioxidant enzymes [35, 36]. On the other hand, HMGB-1, secreted out of the cell with the triggering of damage at the cellular level, can stimulate other cells and cause inflammatory cytokines to be synthesized and released [37]. In addition, it has been shown that HMGB-1 gene expression, whose inhibition is eliminated by SIRT-1 reduction, triggers inflammation in neuroinflammation [38]. As a result of our study, the protective effect of increased SIRT-1 expression with FLV treatment by reversing the levels of both genes may be interpreted as one of the mechanisms of the neuroprotective effect of FLV. Parallelism of both histopathological and immunohistochemical findings and also oxidative stress parameters and gene expressions indicates that FLV treatment is an active substance that should be further investigated in the future. We determined that in the injury group, there was a decrease in SIRT-1 and increased expression of the HMGB-1 gene, which was inhibited, leading to the development of inflammation and oxidative stress due to the decrease in GPX-4. The protective effect of increased SIRT-1 expression with FLV treatment by reversing the levels of both genes may be interpreted as one of the mechanisms of the neuroprotective effect of FLV.

## Conclusion

Parallelism of both histopathological and immunohistochemical findings and also oxidative stress parameters and gene expressions indicate that FLV treatment is an active substance that should be further investigated in the future.

## Data Availability

No datasets were generated or analysed during the current study.
